# CTN-0138: adaptation, implementation, and cluster randomized trial of a Community Pharmacy-Based Prescription Drug Monitoring Program Opioid Risk Assessment Tool—a protocol paper

**DOI:** 10.1186/s13722-024-00514-1

**Published:** 2024-11-18

**Authors:** Gerald T. Cochran, Jennifer L. Brown, Ziji Yu, Adam J. Gordon, Stacey Frede, Clinton Hardy, Melissa Castora-Binkley, Felicity Homsted, Lisa A. Marsch, August F. Holtyn, T. John Winhusen

**Affiliations:** 1 Department of Internal Medicine, University of Utah, Salt Lake City, UT USA; 2 Department of Psychological Sciences, Purdue University, West Lafayette, IN USA; 3 Kroger Pharmacy, Cincinnati, Ohio USA; 4 Pharmacy Quality Alliance, Cincinnati, Ohio USA; 5 Department of Psychiatry, Dartmouth College, Hanover, MD USA; 6 National Institute on Drug Abuse, Cincinnati, Ohio USA; 7 Department of Psychiatry & Behavioral Neuroscience, University of Cincinnati, Cincinnati, Ohio USA; 8 Center for Addiction Research, University of Cincinnati College of Medicine, Cincinnati, Ohio USA

**Keywords:** Opioids, Community pharmacy, Prescription drug monitoring program, Clinical decision support

## Abstract

**Background:**

As the opioid epidemic continues to have a major negative impact across the US, community pharmacies have come under scrutiny from legal systems attempting to hold them accountable for their role in over dispensing and lack of patient intervention. While the most available tool for monitoring patients’ opioid use is Prescription Drug Monitoring Programs (PDMP), these do not provide pharmacists with actionable information and decision support. Our study addresses this gap through three objectives: [1] incorporate validated opioid risk metric thresholds into a PDMP platform to create the *Opioid Risk Reduction Clinical Decision Support* (ORRCDS) tool; [2] assess ORRCDS’ ability to reduce patient opioid risk; [3] assess ORRCDS’ sustainability and viability for broader dissemination in community pharmacy.

**Methods:**

For objective 1, our team is partnering with leadership from the largest US PDMP organization and a top-five pharmacy chain to implement ORRCDS into the pharmacy chain’s workflow following the Guideline Implementation with Decision Support (GUIDES) framework. For objective 2, our team will conduct a type-1 implementation mixed methods study using a 2-arm parallel group clustered randomized design. We anticipate enrolling ~ 6,600 patients with moderate and high opioid use risk during the 6-month enrollment phase across 80 pharmacies. This sample size will provide 96.3% power to detect a 5% or greater difference in responder rate between the intervention and control arm. Responders are patients with moderate-risk at baseline who reduce to low-risk or those with high-risk at baseline who reduce to moderate or low-risk at 180 days post last intervention. To accomplish objective 3, we will use the Consolidated Framework for Implementation Research (CFIR) to develop and execute cross-sectional qualitative interviews with pharmacists (*n* = 15), pharmacy leaders (*n* = 15), and PDMP leaders (*n* = 15) regarding long term adoption and sustainability of the ORRCDS tool.

**Conclusions:**

A PDMP tool that addresses moderate- and high-risk opioid use is not available in community pharmacy. This study will implement ORRCDS in a large retail pharmacy chain that will include additional screening and guidance to pharmacy staff to address risky opioid medication use. Our results will make critical advancements for protecting patient health and addressing the opioid epidemic.

**Supplementary Information:**

The online version contains supplementary material available at 10.1186/s13722-024-00514-1.

## Introduction

The opioid epidemic continues to have a major negative impact across the United States (US; [1]), with overdose rates having significantly increased during the COVID-19 pandemic [2]. Despite shifts in attention on illicitly produced and distributed opioids (e.g., fentanyl), prescribed opioid medications alone or used in combination with synthetic opioids continue to be involved in thousands of overdose deaths annually [1]. Opioid medication misuse is a significant predictor of subsequent overdose [3, 4]. More than 36% of the more than 9 million patients in the US who misused opioid prescriptions (i.e., use of opioid pain medication in a way other than prescribed [5]) in 2021 obtained them through receipt of prescribed medications dispensed through legal means [6]. Overdose deaths from prescription opioids (e.g., oxycodone, hydrocodone) continue to be the second most common source of opioids involved in overdose nationally, with nearly 12,000 deaths in 2022 [7]. Our previous research has consistently demonstrated that 20–40% of patients dispensed opioid medications at community pharmacy settings are engaged in opioid medication misuse [8–10].

Community pharmacies have recently come under close scrutiny from legal systems attempting to hold them accountable for their role in the opioid epidemic for both over dispensing and lack of patient intervention [11–13]. This scrutiny has resulted in large financial penalties and injunctions for practices to improve opioid medication dispensation safeguards and patient intervention services [11–13], such as strengthening prescription drug monitoring, improving medication theft prevention, and limiting oversupplying patients with medications [14]. These actions signal that, in the coming decades, community pharmacies will be required to make significant changes in their policies, workflows, and approaches related to opioid dispensation, stewardship, and patient education.

In addition to the legal impetus pushing pharmacies to greater action related to opioid medication stewardship, a number of benefits exist for these settings that make them a highly attractive location for patient engagement and intervention related to medication misuse reductions. Community pharmacists are among the most accessible health care professionals in the US, with a large majority (more than 90%) of Americans living within *≤* 5 miles of a community pharmacy [15], with many of these locations (more than 40%) having onsite private counseling rooms [16]. Importantly, our previous research has shown that patients are open to pharmacists asking about and discussing their opioid medication use [17].

Given the continued and worsening opioid epidemic, legal exigencies faced, and potential for significant positive impact; it is critical to devise, implement, and test evidence-based strategies and tools to address opioid-related concerns among patients dispensed these medications in pharmacy settings. While the most common tool available to pharmacists for monitoring patients’ opioid use is Prescription Drug Monitoring Programs (PDMP; [18–25]—which have shown positive results for reducing prescribing and limiting days of misuse [19–25]—the efficacy of PDMP alone is unclear regarding its impact on substance use outcomes [22], including rates of overdose [26–28]. A key limitation of PDMPs is the lack of clear and actionable information and decision support available to health care professionals, including pharmacists.

To address these critical gaps in evidence and effective tools, our team proposed, designed, and executed a National Drug Abuse Treatment Clinical Trials Network (CTN) study: *CTN-0093: Validation of a Community Pharmacy-based Prescription Drug Monitoring Program Risk Screening Tool* (i.e., “PharmScreen” [10, 29]). In this project, we collaborated with the largest US PDMP vendor to implement a one-group, cross-sectional health assessment within 19 pharmacies of a top-five largest US chain. The purpose of this assessment and related analyses was to identify clinically meaningful risk thresholds and validate a PDMP-based *opioid risk metric*. The opioid risk metric is an algorithm that weights and sums individual scores for opioid dosages (morphine equivalents), overlapping benzodiazepines and opioid medications, overlapping opioid medications, and numbers of prescribers and pharmacies utilized by patients for opioid dispensing over the last 60, 365, and 730 days—which results in a 2-digit score ranging from 0 to 99. A third digit of the number of active opioid medication prescriptions (1–9, 9 representing 9 or greater prescriptions) is affixed to the end of the score to constitute the full 3-digit score [10, 29]. The gold-standard against which the opioid risk metric was compared was the World Health Organization (WHO) Alcohol, Smoking, and Substance Involvement Screening Test (ASSIST [30, 31]). Study results showed fair concurrent validity for this metric (Area Under the Curve ≥ 0.70; Kappa = 0.35; Spearman correlation = 0.37, *p* < 0.001) and identified low, moderate, and high-risk thresholds anchored to WHO ASSIST risk levels [10].

Having completed this vital groundwork, our team was positioned to move forward with the next steps of (a) implementing the identified risk thresholds into the PDMP platform and then (b) developing a clinical decision support tool for pharmacists. The tool will enable and empower pharmacists to act on the opioid risk metric information. This paper describes the protocol for *CTN-0138: Adaptation and Implementation of a Community Pharmacy-Based Prescription Drug Monitoring Program Opioid Risk Assessment Tool* (i.e., PharmTool; NCT05706311). The PharmTool study has three objectives. The first objective is to implement the opioid risk metric thresholds into the PDMP platform and adapt the platform to create the Opioid Risk Reduction Clinical Decision Support (ORRCDS, pronounced “Orchids”) tool (referred to as ORRCDS or the “tool” throughout this paper). The second objective is to assess the ability of ORRCDS to reduce patient opioid risk. Assessing ORRCDS’ sustainability and viability for broader adoption and dissemination within large-scale pharmacy and corporate environments is the third objective. This paper describes the key design considerations associated with each objective.

## Methods

### Objective 1: Metric implementation and PDMP adaptation

***Design.*** To accomplish the first objective of implementing the opioid risk metric thresholds into the PDMP platform and adapting the platform for intervention delivery, our investigative team is collaborating with leadership from the PDMP partner organization as well as the chain pharmacy organization to incorporate the risk metric thresholds and adapt the PDMP platform for intervention delivery (in the same chain pharmacy corporation mentioned above from CTN-0093). This objective is specifically following the Guideline Implementation with Decision Support (GUIDES) Checklist [32]. The development of GUIDES was originally motivated by the significant potential of decision support tools for implementation of evidence-based practice, yet there have been limited outcomes in previous research literature in terms of use, sustainability, and patient impact [33–35]. Given the complexity of clinical decision support systems, GUIDES was developed by an expert panel to provide structured steps to follow for the development, implementation, and monitoring of decision support tools [32]. GUIDES has four steps, which include: [1] examining the context [2], organizing content [3], ensuring high quality user access, and [4] implementing the ORRCDS tool (see Table 1). Note, steps one and two were targeted for completion within the first ~ four months of the project; step three was targeted for completion within the first ~ 10 months of the project, and step four was targeted for completion within the first ~ 18 months of the project.

**Fig. 1:**
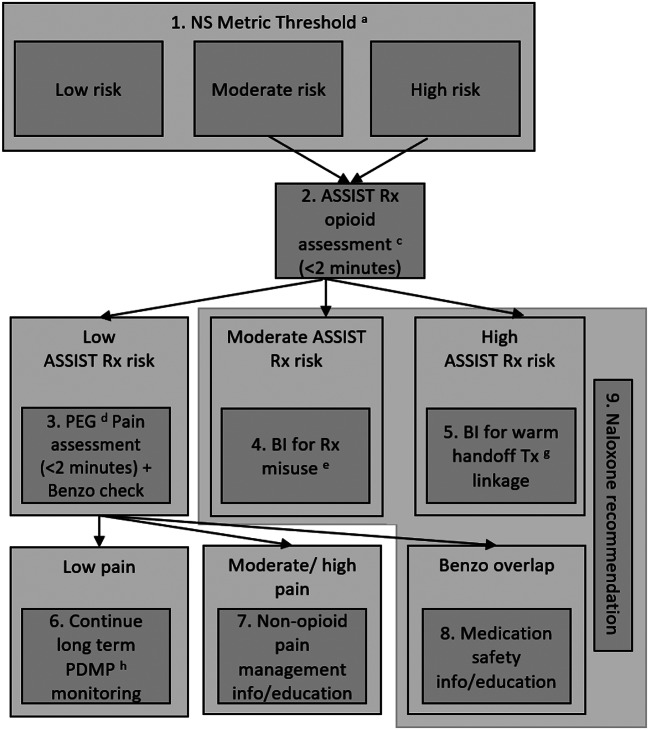
ORRCDS tool flow chart. ^a^ Narcotic score. ^b^ This flow chart only applies to Rx opioid risk. Non-Rx opioid substance screening may be feasible under other circumstances. ^c^ WHO Alcohol, Smoking, and Substance Involvement Screening Test prescription opioid use risk assessment. ^d^ Pain, Enjoyment, General Activity. ^e^ Brief intervention for prescription misuse. ^f^ Brief intervention for treatment linkage. ^g^ Treatment. ^h^ Prescription Drug Monitoring Program

The *examining the context* step of GUIDES involved working with the partnering chain pharmacy to assess existing clinical workflow. To do this, we collaborated with compliance and clinical program development pharmacy corporate leaders who understand regulatory and clinical operations related to opioid dispensation to identify optimal opportunities for when the ORRCDS is triggered in workflow. This workflow assessment allowed tool planning to balance optimizing patient management while accommodating practice demands of pharmacists and technicians. Lastly, this step involved working with the PDMP partner as well as the pharmacy compliance and clinical program development leaders to determine actual workflow within the ORRCDS itself for staff use, see Fig. 1.

**Table 1:** GUIDE framework areas of outcome assessment

Domain	Outcome
Domain 1: Context	Tool can achieve the defined quality objectives
	The quality of the patient data is adequate
	Stakeholders and users accept tool
	Tool can be added to the existing workload, workflows and systems
Domain 2: Content	The content provides trustworthy evidence-based information
	The decision support is relevant and accurate
	The decision support provides an appropriate call to action
	The amount of decision support is manageable for the target user
Domain 3: High quality user access	The system is easy to use
	The decision support is well delivered
	The system delivers the decision support to the right target person
	The decision support is available at the right time
Domain 4: Implementation	Information to users about the tool system and its functions is appropriate
	Other barriers and facilitators to compliance with the decision support advice are assessed/addressed
	Implementation is stepwise and the improvements in the tool are continuous
	Governance of the tool implementation is appropriate

The *organizing content* step of GUIDES involved our study investigators as well as pharmacy chain partners laying out all of the ORRCDS content. Briefly (see greater details in the intervention section below), ORRCDS will categorize patients into low, moderate, and high risk. Moderate- and high-risk status based on risk metric scores will be confirmed by a brief self-report assessment (i.e., the ASSIST prescription opioid risk subscale; [30, 31]. These measures were selected given their clinical utility while at the same time being able to accommodate the pharmacy locations and related demands in terms of brevity. False positive patients will be assessed for pain (i.e., the Pain, Enjoyment of Life and General Activity scale [PEG] [36]) and provided resources. Moderate risk patients will be provided with a brief motivational intervention to reduce opioid risk behaviors and given resources—including naloxone referral. High risk patients will be provided with a warm handoff to their primary care provider delivered in the style of a brief motivational intervention to discuss addiction and other treatment options. These patients will also be provided a naloxone referral for a kit to be mailed to them free of charge (see Fig. 1). The content for each risk level was created by developing scripts utilizing brief motivational intervention principles coupled with normative feedback.

The *ensuring high quality user access* step included adapting the existing PDMP platform in collaboration with PDMP and pharmacy partners to ensure screening, motivational intervention/feedback, naloxone recommendation, and warm handoff/treatment linkage tools are easy to use, well-delivered, and activate at optimal times. This has involved an iterative approach with the investigative team presenting ideas and designs for ORRCDS, incorporating those ideas and designs into mock-up slides, reviewing these slides with investigators and pharmacy/PDMP leaders, and then revising the mock-up slides based on feedback.

The final step is *implementing the tool*, which entails two parts. The first is actual tool implementation within the PDMP and pharmacy chain systems. ORRCDS implementation followed the pharmacy chain standard 3-phase approach: (1) pilot site initiation over 1 month; (2) receive feedback from staff/make needed adjustments; and (3) full study testing thereafter. The second part established a training module with the chain pharmacy required for pharmacy staff before site initiation of the intervention (see details below).

***Measurement and analysis***. Objective 1 will be assessed using the GUIDES evaluation framework. This framework includes 4-items per above specified domains, each with a 7-point Likert scale for scoring [32]. This tool will be administered to study stakeholders including investigators, corporate leaders from pharmacy sites and PDMP partners, and a National Institute on Drug Abuse Scientific Officer (assigned to the project by virtue of the cooperative agreement funding mechanism). We will also utilize the System Usability Scale, a brief 10 item measure of perceived computer-based program usability [37, 38], to assess the yes/no go-live readiness of ORRCDS. This assessment will be administered to pilot pharmacy site staff who utilize the tool.

Frequencies, percentages, and measures of central tendency will be employed to assess results from the GUIDES responses. Specifically, each of the above Likert scale items will be described using mean and standard deviation as well as median and interquartile range. For the supporting items, which are yes/no responses (not shown herein), we will calculate frequencies and percentages to characterize responses to these items. For the System Usability Scale, we will summarize the overall score. A score of 68 or higher is considered the cut off for yes/no usability [39, 40]. Having the subjective GUIDES assessment as well as the objective System Usability Scale provides rich insights for the investigative team into the readiness of ORRCDS and avenues needed for improvement before full trial launch.

### Objective 2: Testing the ORRCDS

***Design***. To accomplish the second objective of this study of assessing the impact of the ORRCDS tool on patient opioid risk, our study team will conduct a type-1 implementation mixed methods study using a 2-arm parallel group clustered randomized design. This design clusters patients within pharmacy sites, randomized on a 1-to-1 basis to the ORRCDS vs. usual care. We chose the cluster design given its advantages over patient-level randomization and the length of time required by stepped-wedge designs. Regarding patient level randomization, given the real world application of this tool, training staff to deliver the intervention to only some patients within sites and not others would create a greater risk for condition contamination compared to randomizing by site. The cluster design will reduce the possibility of patients being exposed to both conditions, thus preventing contamination. Regarding length of time, a stepped-wedge design that would attempt intervention rollout in 80 busy pharmacy sites would require a prohibitive amount of time for site intervention delivery initiation, intervention utilization, and follow up.

**Fig. 2:**
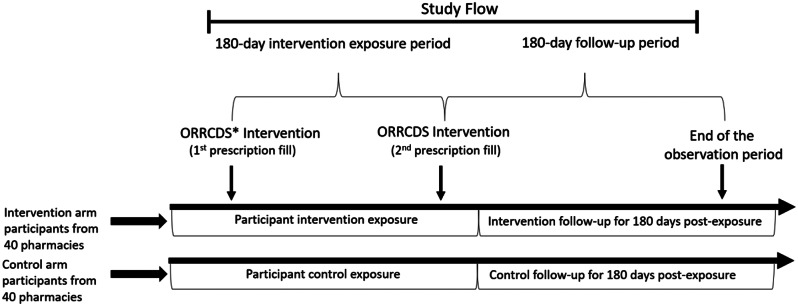
Study design. ^*^ Opioid risk reduction clinical decision support

The randomization will be stratified by zip code poverty level and total pharmacy volume of medication dispensed. Stratification by dispensing volume will ensure an even distribution of higher vs. lower volume stores in each condition—given that high volume sites may be more likely to not intervene with each patient given more intense workload. Advantages of site- vs. patient-level randomization include strengthened internal validity from mitigation of potential condition contamination as well as lower costs—given the number of sites and patients involved herein. ORRCDS will be evaluated within two divisions of a pharmacy chain (*n* = 40 intervention pharmacies/*n* = 40 usual care pharmacies) in the state of Ohio. This design includes a 6-month enrollment phase and a 6-month follow up observation phase (see Fig. 2).

***Study population***. Data from patients within pharmacy clusters will be utilized for analysis. Those patients prescribed an opioid medication, seeking opioid prescription dispensation in a study pharmacy, ≥ 18 years of age, and who have an opioid risk metric of moderate or higher will be included in the study cohort. Those solely receiving buprenorphine formulations for opioid use disorder treatment, without an additional dispensation of a pain medication during the study intervention exposure period or those that received the majority (more than 50%) of their opioid medication dispensations (other than buprenorphine) from non-study pharmacies will be excluded. These data will be obtained from the PDMP database for the state based on a waiver of consent approval from the University of Utah Institional Review Board.

***Interventions***, ***training***, ***and supervision***. Discussed briefly above, the ORRCDS condition involves ORRCDS alerting the pharmacist during the drug utilization review process that a potentially at-risk patient has an opioid medication in queue for pickup; these alerts are based on the previously identified thresholds from the PharmScreen preliminary study [10]. Patients identified as moderate or high risk would receive confirmatory opioid misuse screening. Those identified as low-risk on confirmatory screening will receive auxiliary pain screening on the PEG [36]. Patients reporting moderate/high pain with low opioid risk will receive non-opioid pain management information/education [41]; such education may increase the utilization of non-opioid pain management strategies [42].

Patients with moderate risk on confirmatory opioid misuse screening will receive a brief motivational intervention incorporating normative feedback that targets behaviors associated with opioid misuse (e.g., early refills of opioid medication, seeking multiple opioid prescriptions) coupled with naloxone recommendation and referral. The ORRCDS tool is designed to assist pharmacists in systematically assessing and intervening in cases of risky prescription opioid use and management. This tool will guide the pharmacist through an evaluation of the patient’s risk factors and potential barriers to change, employing motivational interviewing techniques to engage the patient in exploring safety driven behavior change. The risk factors targeted for intervention include, as listed above, opioid dosages, overlapping benzodiazepines and opioid medications, overlapping opioid medications, and numbers of prescribers and pharmacies utilized by patients. Based on the assessed risk level, the tool facilitates a warm handoff to primary care and encourages the patient and primary care provider to discuss a connection to specialized treatment when possibly needed. This approach aims to effectively address opioid misuse while fostering patient-centered care.

In at least four separate clinical trials (*N* = 32, *N* = 62, *N* = 126, *N* = 204 [9, 43–45]) with patients receiving brief motivational interventions to reduce opioid medication misuse/misuse behaviors, study results showed significant improvements [9, 43–45]. In either scenario of moderate or high risk, the pharmacist’s goal is to assist the patients in making health behavior changes to reduce risk—the goal is not to deny or take away access to opioid medication treatment, which could result in detrimental unintended consequences [46–48].

Those with confirmed high risk will receive a warm handoff delivered in the style of a brief motivational intervention to connect patients by phone to their primary care provider to discuss substance use treatment or other treatment needs (such as pain management). These patients likewise will receive a naloxone referral. A rich literature shows warm handoff is an evidence-based method for connecting individuals with substance use needs to treatment [49–53]. Patients with elevated risk will receive up to 2 intervention sessions over the course of up to 2 dispensation encounters with pharmacists—interventions will be similar each session if risk status does not change between encounters. The maximum duration for an opioid prescription in Ohio is 90 days.

ORRCDS training will involve pharmacists at the intervention pharmacy sites being emailed brief reading materials regarding the foundational work of this project. Next, a series of web-based, pre-recorded trainings that instruct pharmacists on using the tool will be made available via an electronic education platform. Pharmacists will be required to view the videos to familiarize themselves with the required steps of the intervention. The prereading and recorded videos each last approximately one hour for a total of two hours. Post tests will be administered in the education platform to ensure material comprehension and retention. Training completion/ attendance will be captured by the training platform.

The study team will also provide monthly supervision sessions during the 6-month intervention period. All intervention site pharmacists will be required to attend at least three of these zoom/telephone sessions to receive booster training from study staff and engage in question-and-answer discussions. Sessions will include didactic material, session examples, and question and answer periods. These supervision sessions will last approximately 30 min and attendance will be captured by study staff leading these sessions.

Intervention delivery performance monitoring will take place weekly by the PDMP vendor providing numbers regarding how many interventions were delivered at unique pharmacies along with the total number of patients in that week who received opioid medications. This will allow the study team to monitor total intervention opportunities and compare those to the actual number delivered and to allow the study investigators to provide site feedback and encouragement in the utilization of the tool.

For the usual care condition, pharmacists are required to perform a universal PDMP review before initial dispensations [54]. Pharmacists are also required to offer brief counseling (e.g., unstandardized information about medication safety) for new/modified prescription therapies [55]. Pharmacists are trained and monitored (via dispensing record system alert) for these requirements by the chain pharmacy partner.

***Outcomes and data collection.*** Study data collection will follow the Reach, Effectiveness, Adoption, Implementation, and Maintenance (RE-AIM) implementation science evaluation framework. Table 2 shows the specific indicators that will be employed in this study, ordered by primary/secondary outcomes [56]. Given the type-1 focus of this study, the primary efficacy outcome will be assessing changes across time for patients’ opioid risk metric level from high to moderate risk or moderate to low risk, see power calculation below. Mentioned above, this metric was validated in CTN-0093, using the WHO ASSIST [10]. Secondary efficacy outcomes will include possible improvements in measures of opioid utilization over time, such as changes in performance on opioid safety measures developed by the Pharmacy Quality Alliance including high opioid dose (greater than or equal to 90 morphine milligram equivalents over 90 days) and overlapping benzodiazepine use (see Table 2 [57–59]).

***Sample size and power.*** We anticipate that ~ 6,600 (based on CTN-0093 pharmacy site estimates) moderate/high-risk patients (evaluated by the opioid risk metric) will be enrolled during the 6-month enrollment phase across the 80 pharmacies. The power calculation is presented for multiple scenarios with different assumptions of intraclass correlation (ICC) and effect size (Table 3). The proposed sample size will provide 96.3% power with 2-sided $\:{\upalpha\:}$= 0.05 to detect a 3% or greater difference in responder rate between the intervention arm and control arm. Responders are defined as patients with moderate-risk at baseline who reduce to low-risk or those with high-risk at baseline who reduce to moderate or low-risk at 180 days post last intervention. Risk categories are defined by the thresholds identified in CTN-0093, mentioned above [10]. The power consideration assumes an ICC of 0.05, typical for clustered randomized trials of patient’s outcomes [60, 61]. Importantly, the numbers of pharmacies chosen in this study approximates that of a division within the pharmacy chain. Thus, the results herein will have a real-world reference size within the chain pharmacy corporation.

**Table 2:** Reach, effectiveness, adoption, implementation, and maintenance implementation science evaluation framework construct chart

Component	Metric	Data source	Assessment timing	Outcome
Effectiveness	Narcotic Score	PDMP	Preintervention, intervention, postintervention	Primary efficacy
	Fatal overdose	Death certificates	Preintervention, intervention, postintervention	Secondary efficacy
	High dose (PQA)	PDMP	Preintervention, intervention, postintervention	Secondary efficacy
	Multiple providers (PQA)	PDMP	Preintervention, intervention, postintervention	Secondary efficacy
	Overlapping benzo (PQA)	PDMP	Preintervention, intervention, postintervention	Secondary efficacy
	Morphine equivalents	PDMP	Preintervention, intervention, postintervention	Secondary efficacy
	Buprenorphine uptake	PDMP	Preintervention, intervention, postintervention	Exploratory efficacy
	# patients approached	CDS tool	Intervention	Primary implementation
Reach	CDS tool use	CDS tool clicks	Intervention	Primary implementation
Implementation	Perceptions CDS barriers	Pharmacists	Pre/post intervention	Secondary implementation
Adoption	Perceptions CDS barriers Disposition on continued CDS use	Pharmacist partner leader interviews	Pre/post intervention	Secondary implementation
		Pharmacist partner leader interviews	Pre/post intervention	Secondary implementation
Maintenance	Disposition on continued CDS use	PDMP partner leader interviews	Pre/post intervention	Secondary implementation

**Table 3:** Power calculation with different assumptions for intra class correlation and effective size

ICC	Design Effect^a^	Total *N*	Effective *N* per arm^b^	Effective Size^c^	Power
0.01	2.24	6600	1471	10%	100
0.01	2.24	6600	1471	8%	100
0.01	2.24	6600	1471	5%	100
0.01	2.24	6600	1471	3%	100
0.01	2.24	6600	1471	1%	97.1
0.03	4.72	6600	698	10%	100
0.03	4.72	6600	698	8%	100
0.03	4.72	6600	698	5%	100
0.03	4.72	6600	698	3%	99.6
0.03	4.72	6600	698	1%	75.5
0.05	7.20	6600	457	10%	100
0.05	7.20	6600	457	8%	100
0.05	7.20	6600	457	5%	100
0.05*	7.20	6600	457	3%	96.3
0.05	7.20	6600	457	1%	57.3
0.1	13.43	6600	246	10%	100
0.1	13.43	6600	246	8%	99.6
0.1	13.43	6600	246	5%	94.7
0.1	13.43	6600	246	3%	78.3
0.1	13.43	6600	246	1%	34.9

^a^Design effect: this is a correction factor that is used to adjust the required sample size due to the clustered randomized design. If a simple randomization is used and the required sample size is N, then to achieve the same power using the clustered randomized design, the sample size should be N times Design effect

^b^Effective *N* per arm: the required sample size to achieve the same statistical power if a simple randomization design is used instead of cluster randomized design. Effective sample size per arm * 2 * design effect = Total N for clustered design

^c^Effective size: the anticipated difference between the 2-intervention arm in responder rate

***Analysis plan***. The primary efficacy endpoint is the responder rate at 180 days post final intervention. We will fit a generalized linear mixed model to relate the primary outcome (i.e., responder status defined based on opioid risk metric) by treatment arm, controlling for baseline covariates including but not limited to the stratification factors, baseline risk level assessment, and other relevant/available patient characteristics, including patient age, sex, insurance status, and location using pharmacy zip code. To model the dichotomous response variable, we will employ the binary distribution with logit link. The random intercept and the unstructured variance covariance matrix will be used to account for within pharmacy correlation. If the model fails to converge, the compound symmetry structured variance covariance matrix will be used.

### Objective 3: Assessment of long-term sustainability and viability

***Design and target population.*** To accomplish the third study objective of assessing ORRCDS’s sustainability and viability for broader adoption and dissemination within large-scale pharmacy and corporate environments, our study team will execute cross-sectional qualitative interviews with pharmacists from the chain partner as well as non-partner pharmacists (*n* = 15), leaders from the chain pharmacy partner (*n* = 15), and leaders from the PDMP company (*n* = 15). Participants will be identified by referral from the pharmacy and PDMP partners as well as through outreach to professional licensing lists of pharmacists in the state of Ohio. Interviews will be designed to explore barriers and facilitators of long-term adoption and sustainability of the implemented tool within the pharmacy and PDMP practice and corporate environments. Interviewees will be selected using a purposive sampling approach. To be included as a participant in this interview, individuals must be a licensed pharmacist, pharmacy leader, or leader within the PDMP company and believe they can provide insights and opinions regarding the long-term adoption and sustainability of the tool.

***Interview guide.*** We will employ the Consolidated Framework for Implementation Research (CFIR) as the foundation for the qualitative interview portion of this study [62, 63], see Supplement 1. The CFIR is a multidimensional model consisting of standardized constructs for understanding and approaching objectives across the continuum of implementation science [64], ranging from pre-implementation activities to post-implementation outcome analyses [65–67]. The CFIR has an extensive literature base for formulating and implementing evidence-based interventions in real-world practice [64]. The CFIR has been applied across a number of programmatic areas, including health care delivery, health care processes redesign, quality improvement, health promotion, and disease management on topics such as mental health, obesity, and high blood pressure [64].

We will employ the CFIR interview guide for qualitative research to assess barriers to, and feasibility of: ORRCDS use in pharmacy settings, system-level adoption and implementation, and continued tool use [62]. Items will be selected by the research team in collaboration with the chain pharmacy leadership and in collaboration with PDMP partners. Interviews will also explore pharmacist perception of ORRCDS acceptability by patients. Examples of topic areas to be queried will include perceived feasibility of ORRCDS utilization within the retail pharmacy practice environment. Interviews will further explore what challenges exist that could impede adopting ORCCDS, i.e., getting the tool into practice within the broader partner pharmacy system or pharmacies outside the partner system. Interviews will also explore what characteristics, conditions, or outcomes need to be presented to pharmacists and corporate leaders that will ensure long term consistent use of ORRCDS.

***Qualitative sample size***. Previous research has demonstrated sample sizes of 20 individuals are sufficient to achieve thematic saturation between the researcher and participants, which will generate rich data for elucidating complex relationships [68]. Previous research has also demonstrated samples of approximately 12 interviews can reach saturation of findings [69]. Saturation has been defined as a point beyond which no significantly new information is being obtained. Lincoln and Guba’s framework [70] will be used to address and meet criteria for quality and rigor in this study and involve credibility, dependability, confirmability, and transferability [70].

***Qualitative analyses***. For the qualitative data, we will follow methods recommended by Braun and Clarke to capture associations between categories and extract and conceptualize themes [71]. We will independently review interview data and code inductively and deductively. Codes will be clustered based on their similarities into categories. Our team will meet multiple times to discuss the emergent themes [71]. Coded data will be described using frequencies and percentages.

## Discussion

The opioid epidemic continues to have serious negative repercussions for individuals across the US. Despite the broad availability of community pharmacies, high level of trust for pharmacists, and unparalleled training and knowledge regarding medication management, these settings have had limited utilization in addressing the opioid epidemic. This study is positioned to advance the field in 3 specific areas: (1) This study has the potential to drastically advance involvement of community pharmacy in addressing the national opioid epidemic. (2) The model and tool implemented in this study have the potential for national scalability. (3) Outcome metrics in this study have high external validity.

### Pharmacy involvement in the opioid epidemic

While community pharmacy has been at the center of the opioid epidemic for several decades in dispensing opioid medications that are subsequently misused, it has only recently become a focus as a potential site for solutions. A few academic research teams have begun to push forth behavioral [9, 72], overdose reversal medication distribution [73–77], and even pharmacy delivery and management of medications for opioid use disorder [78, 79]. While progression into proactive solutions to address the epidemic has been embraced by some chains, independent, and health system pharmacies—most are moving slowly while some are primarily responding to injunctions placed upon them as part of legal proceedings [11–13]. Indeed, this current study sets a crucial stage for future opioid use disorder care/management models within community pharmacy settings in the coming years.

### Scalability

The pharmacy and PDMP partners involved in this study are among the largest in the US. Mentioned above, previous research that has attempted forays into opioid interventions/ treatments within community pharmacy settings have done so often within independent/small-scale settings. While these efforts have been paramount for demonstrating proof of concept, acceptability, feasibility, and preliminary efficacy—few have been executed within nationally scalable environments. Taking interventions to scale within any environment is highly complex with several inherent challenges [80, 81]—not the least of which is an environment with the capacity to scale-up and scale-out services. Both the pharmacy chain organization and the PDMP organization partners in this study are regularly developing, testing, and implementing new aspects of patient care and information systems. Thus, the partnership with these entities for this project has the potential to result in strategic positioning for broader dissemination and implementation following successful study completion. However, noted within the original validation study of the opioid risk metric, this measure’s validation was limited to a largely white, homogeneous sample within the US Midwest [10]. This current study is being conducted in the same region of the US; these limitations must be taken into account when attempting to implement these tools in other regions of the US with more heterogeneous populations.

### Real world/high external validity metrics

One of the significant challenges in the field for the past several years has been describing and defining opioid use and misuse within administrative data in consistently reliable and valid ways. Some studies have attempted to provide greater parameterization and operationalization of measures of opioid use [82, 83]. These efforts have been critical to enhance the external validity and comparability of populations across studies and settings. Organizations like the Pharmacy Quality Alliance have developed a number of these metrics in an effort to bring uniformity in measurement of opioid use and misuse across systems and payers [57–59]. By employing such metrics in this project to illustrate changes experienced by the patients exposed to the intervention condition, our results will have important translation to other settings for possible benchmarking comparisons in possible changes to risk opioid use.

## Conclusion

A PDMP-based tool that addresses moderate and high-risk opioid use is not widely available in community pharmacy. This study will implement the ORRCDS in a large retail pharmacy chain that will include additional screening and guidance to pharmacy staff on how to provide brief misuse intervention, naloxone dispensation, and warm handoff. Such steps addressing risky opioid medication use will make critical advancements for protecting patient health and addressing the national opioid epidemic.

## Electronic supplementary material

Below is the link to the electronic supplementary material.

Supplementary Material 1

## Data Availability

Not applicable.

## Abbreviations

US: United States
PDMP: Prescription Drug Monitoring Programs
CTN: National Drug Abuse Treatment Clinical Trials Network
WHO: World Health Organization
ASSIST: Alcohol, Smoking, and Substance Involvement Screening Test
ORRCDS: Opioid Risk Reduction Clinical Decision Support
GUIDES: Guideline Implementation with Decision Support
PEG: Pain, Enjoyment of Life and General Activity scale
RE-AIM: Reach, Effectiveness, Adoption, Implementation, and Maintenance
ICC: Intraclass correlation
CFIR: Consolidated Framework for Implementation Research
