# Supplementary material for: CTN-0138: adaptation, implementation, and cluster randomized trial of a Community Pharmacy-Based Prescription Drug Monitoring Program Opioid Risk Assessment Tool—a protocol paper
Source: Addict Sci Clin Pract. 2024 Nov 18;19:82. doi: 10.1186/s13722-024-00514-1 (PMC11572521; doi:10.1186/s13722-024-00514-1)
Supplement: Supplementary file 1 — Supplementary Material 1 [file 13722_2024_514_MOESM1_ESM.docx]

**Supplement 1: PharmTool Qualitative Interview Guide**

1. Could you please tell me a little about your role at your company and your experience in community pharmacy?
2. What do you believe are the needs and preferences of patients who are prescribed an opioid and may be involved in misuse (use outside of what is directed by their doctor)? Why do you think this? Can you share a specific example or situation?

[*Description of the intervention tool.*] The following questions will ask your opinions about implementing an opioid tool like ORRCDS at your pharmacy.

1. How well do you think an opioid risk-reduction tool for patients who use prescribed opioids will meet the needs of the patients served by chain pharmacies? Why do you think this?
2. What barriers do you anticipate in providing this intervention? What are the barriers to patients accepting the service? Why do you think this?

The following questions are about how you think pharmacy staff or leaders perceive the current state of use of opioids as intolerable or in need of change.

1. In your opinion, what is the level of need for an opioid risk-reduction tool for patients who may misuse prescribed opioids? Why?
2. What type of financial or other incentives for pharmacy staff would influence the decision to implement such a service line? Why?

The following question is about pharmacy staff and leaders’ perceptions of the quality and validity of evidence supporting the belief that the intervention will have desired outcomes.

1. What kind of supporting evidence or proof is needed about the effectiveness of this service line to get pharmacy staff (for example, pharmacists, pharmacy technicians, and pharmacy interns) on board?

The following questions will ask about practical changes possibly needed at the pharmacy to accommodate this intervention for opioids.

1. In your opinion, what types of workflow changes will be needed to accommodate an opioid risk-reduction tool? Why?
2. In your opinion, how can pharmacy software or other technology help implement this opioid risk-reduction tool? Why?

The next couple of questions are about the culture at your organization. Culture, in this case, is the values, general beliefs, and basic assumptions of a given organization.

1. How would you describe the culture of your organization? Of your own setting or unit?
2. How do you think your organization's culture (general beliefs, values, assumptions that people hold) will affect the implementation of the intervention?
3. What aspects of your organizational culture will help maintain long term the use of the intervention tool? What aspects may be barriers?
